# Phenotypic instability and epigenetic variability in a diploid potato of hybrid origin, *Solanum ruiz-lealii*

**DOI:** 10.1186/1471-2229-9-21

**Published:** 2009-02-20

**Authors:** Carlos F Marfil, Elsa L Camadro, Ricardo W Masuelli

**Affiliations:** 1 Laboratorio de Biología Molecular, EEA La Consulta INTA, Facultad de Ciencias Agrarias, Universidad Nacional de Cuyo and CONICET, M5528AHB, Alte. Brown 500, Chacras de Coria Mendoza, Argentina; 2 EEA Balcarce, INTA-FCA, UNMdP and CONICET, CC 276, 7620, Balcarce, Bs. As., Argentina

## Abstract

**Background:**

The wild potato *Solanum ruiz-lealii *Brüch. (2n = 2x = 24), a species of hybrid origin, is endemic to Mendoza province, Argentina. Recurrent flower malformations, which varied among inflorescences of the same plant, were observed in a natural population. These abnormalities could be the result of genomic instabilities, nucleus-cytoplasmic incompatibility or epigenetic changes. To shed some light on their origin, nuclear and mitochondrial DNA of plants with normal and plants with both normal and malformed flowers (from here on designated as plants with normal and plants with abnormal flower phenotypes, respectively) were analyzed by AFLP and restriction analyses, respectively. Also, the wide genome methylation status and the level of methylation of a repetitive sequence were studied by MSAP and Southern blots analyses, respectively.

**Results:**

AFLP markers and restriction patterns of mitochondrial DNA did not allow the differentiation of normal from abnormal flower phenotypes. However, methylation patterns of nuclear DNA discriminated normal and abnormal flower phenotypes into two different groups, indicating that abnormal phenotypes have a similar methylation status which, in turn, was different from the methylation patterns of normal phenotypes. The abnormal flower phenotype was obtained by treating a normal plant with 5-Azacytidine, a demethylating agent, giving support to the idea of the role of DNA methylation in the origin of flower abnormalities. In addition, the variability detected for DNA methylation was greater than the detected for nucleotide sequence.

**Conclusion:**

The epigenetic nature of the observed flower abnormalities is consistent with the results and indicates that in the diploid hybrid studied, natural variation in methylation profiles of anonymous DNA sequences could be of biological significance.

## Background

**Figure 1:**
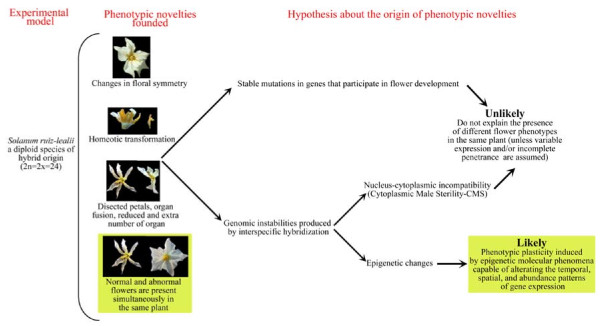
**Diagram showing the experimental plan and the hypothesis tested to explain the phenotypic abnormalities observed**.

In 1962, Brücher described *S. ruiz-lealii *Brüch. (2n = 2x = 24), as a new species from Argentina endemic to Southern Mendoza province [1]. Hawkes and Hjerting [2] considered that *S. ruiz-lealii *was a natural hybrid between *S. kurtzianum *Bitter et Wittm. (2n = 2x = 24) and *S. chacoense *Bitter (2n = 2x = 24). In a recent report based on the analysis of morphological and molecular (SSR markers) data in natural populations of *S. ruiz-lealii *and accessions of the putative parental species from a germplasm bank, Raimondi *et al. *[3] suggested that *S. ruiz-lealii *might not be a recent natural hybrid between *S. kurtzianum *and *S. chacoense *but has probably originated by hybridization between *S. chacoense *and another taxon, or by divergence of *S. chacoense*. In this study, we provide further evidence of the hybrid origin of *S. ruiz-lealii*. Raimondi *et al. *[3] reported high morphological similarity between different accessions of *S. ruiz-lealii*, but also that some plants of this species had notable flower malformations. These malformations could be the result of stable mutations in genes that participate in flower development; however, since both normal and malformed flowers were simultaneously observed in different inflorescences of the same plant and, also, normal and malformed flowers were observed in the same inflorescence, this hypothesis would not be very likely unless variable expression and/or incomplete penetrance of the mutant gene(s) involved are assumed. Less irrevocable processes than mutations could be responsible for the abnormalities observed (Figure 1).

In potatoes, Grun *et al. *[4] detected deformed flowers in the progeny of a backcross (*S. phureja *× *S. chacoense*) × *S. chacoense*. These deformed flowers either lacked anthers or these were present as rudiments. Deformed corollas, slightly shortened styles, and female sterility were sometimes associated with these flowers. It was hypothesized that deformed flowers resulted from the interaction between a single recessive *df *gene present in certain *S. chacoense *plants and effective in the sensitive plasmon [*df*^*s*^] of certain *S. phureja *plants. Also, *S. chacoense *plants with the *Df *allele for normal flower were described [4]. Later, interactions between hypothetical nuclear genes and cytoplasmic factors, leading to alterations in male fertility and flower development in various species combinations, prompted evolutionary studies in *Solanum *spp. [5], and the first attempts to understand the genetic and molecular mechanisms underlying cytoplasmic male sterility (CMS) in this genus [6]. CMS is a maternally inherited loss of male fertility based on flower male organ dysfunctions that range from anthers in a low position or reduction in pollen fertility (the simplest manifestations) to a complete conversion of stamens into other floral organs [7].

Mitochondrial genes have been found to be associated with CMS traits in most plant species so far examined [8]. Comparison of chloroplast DNA in several potato species suggests that the expression of CMS in this group is not controlled by cpDNA [9], as also noted in other species like maize, rice, petunia, sunflower, wheat, *Brassica *and *Phaseolus *[for reviews see [8,10,11]].

Variations in DNA methylation patterns can result in phenotypic instability [12,13]. In plant genomes, cytosine methylation of CpG and CpNpG nucleotides varies in frequency along the chromosome and regulates gene expression either at the gene level or, else, regionally, influencing entire chromosome regions [14]. Ample evidence has been obtained to support this concept, and DNA methylation is now recognized as a chief contributor to the stability of gene expression and chromatin structure. Global analyses of genetic epigenetic and transcriptional polymorphisms in *Arabidopsis thaliana *suggest a possible relationship between natural CG methylation variation and gene expression variation [15]. Several studies have recently reported defects in flower development caused by heritable epigenetic alleles (epialleles) associated with abnormal DNA methylation. Hypermethylated epialleles of *AGAMOUS *[16] and *SUPERMAN *[16,17], which affect flower structure, and hypomethylated epialleles of *FWA *[18], which delay flowering time, have been recovered from both mutagenized *Arabidopsis *populations and DNA hypomethylated lines such as *ddm1*, *met1*, and antisense-cytosine methyltransferase *MET1 *[19,20]. Natural plant epialleles affecting ecologically important traits, like floral symmetry and fruit ripening, have been described [21,22]. Also, a connection between DNA methylation and phenotypic instabilities was demonstrated using demethylating agents such as 5-Aza-2'-deoxycytidine and 5'-Azacytidine [23,24].

The study of nuclear and cytoplasmic genomes as well as DNA methylation variability is important for understanding the basis of phenotypic variation and microevolution in natural plant population and, also, for artificial selection in breeding programs. Wild tuber-bearing *Solanum *species constitute an important reservoir of genetic diversity and resistance/tolerance to biotic and abiotic stresses for potato improvement [25]. However, epigenetic variation is often overlooked as a source of phenotypic variation for artificial selection. Stokes *et al. *[26] reported the molecular mechanism underlying a *ddm1 *induced pleiotropic defect, *bal*, that associates epigenetic regulation and plant pathogen defense responses. In the *bal *variant, overexpression of one gene in the cluster of NBS-LRR-class disease-resistance-genes stimulates the disease response pathway and causes dwarfing and elevated resistance. Also, variation in the methylation status of the *patatin *gene among plants of synthetic hybrids between *S. kurtzianum *and *S. tuberosum *has been reported [27]. Variation in epigenetic information encoded at the chromatin level rather than at the nucleotide sequence level is commonly thought to be transient and unlikely to underlie stable changes in phenotype. There is considerable evidence, however, that epigenetic changes, particularly those due to alterations in DNA methylation, can be inherited through meiosis and mimic traditional mutations [28].

The extent to which epiallelic variation is an important common contributor to phenotypic variation in natural plant populations and its consequences on fitness remain unknown. Thus, the purposes of the present study were: a) to explore the possible origin of flower abnormalities in plants of a wild diploid potato species of hybrid origin, *S. ruiz-lealii*, and b) to evaluate the genetic and epigenetic variability in plants of a natural population. To this end, we examined nuclear and mitochondrial DNA variability as revealed by analyses of amplified fragment length polymorphism (AFLP) and restriction pattern markers, respectively, and the methylation status detected by the methylation sensitive amplified polymorphism (MSAP) technique in plants with normal and plants with both normal and malformed flowers (from now on, referred to as plants with normal and plants with abnormal flower phenotypes, respectively). Neither the mtDNA nor the nDNA polymorphism explained the flower variability observed. The methylation polymorphism detected was higher than the nDNA variability and allowed the grouping of genotypes according their flower phenotype. Finally, we aimed at producing an abnormal flower phenotype from a plant with normal flower phenotype, by treatment with 5-Azacyitidine (azaC).

## Results

### Flower morphology, pollen viability and cytology

**Figure 2:**
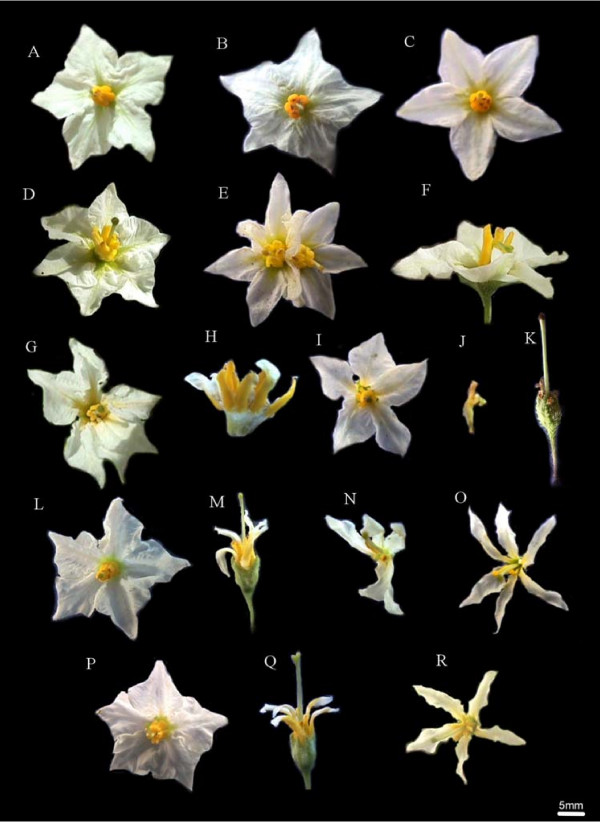
**Flower phenotypes of eight *S. ruiz-lealii *plants**. A, B and C, normal flowers from plants 13.4, V0 and 17.1, respectively. D, E and F, flowers from plant 6. D, intermediate flower phenotype with twisted anthers and normal petals and style. E, twin flower. F, flower with twisted style. G and H, flowers from plant 13.2. G, flower with bilateral symmetry. H, staminoid petals (sepals and style removed). I, flower from plant 19.3 that resembles normal flower, but with dissected petals. J, carpelloid stamens from plant 19.3. K, extreme flower phenotype from plant 19.3, with rudimentary stamens and petals. L, M, N and O, flowers from plant 03. L, normal flower. M, flower with staminoid petals and rudimentary stamens. N, flower with reduced number of stamens, dissected petals and fusion between a stamen and the pistil. O, flower with extra number of petals (dissected) and reduced number of stamens. P, Q and R, flowers from plant 9. P, normal flower. Q, flower with staminoid petals, rudimentary stamens and bifurcated stigma. R, flower with dissected petals and twisted anthers.

The morphology of the leaves, tubers and stolons was normal in all plants studied, but normal and malformed flowers co-existed in each of five plants (Table 1). Pollen viability of normal flowers ranged from 18 to 80%; on the other hand, malformed flowers in those same plants bore no pollen. In Figure 2, the observed flower phenotypes are presented; the corolla shape of normal plants 13.4, V0 and 17.1 varied from rotate (Figure 2A–B) to stellate (Figure 2C). Flower abnormalities found in different plants included: twisted anthers and style and twin flowers (Figure 2D–F); bilateral symmetry and dissected petals (Figure 2G and 2I); rudimentary stamens and petals (Figure 2K); homeotic changes like staminoid petals (Figure 2H, M and 2Q) and carpelloid stamens (Figure 2J).

**Table 1:** Flower phenotype and pollen viability of plants studied.

Plants	Flower types	Homeotic Transformation	Pollen viability (%)	Phenotype classification
19.3	Normal	_	18	Abnormal
	Abnormal	Yes	Without pollen	
				
9	Normal	_	65	Abnormal
	Abnormal	Yes	Without pollen	
				
03	Normal	_	66	Abnormal
	Abnormal	Yes	Without pollen	
				
13.2	Normal	_	56	Abnormal
	Abnormal	Yes	13	
				
6	Normal	_	80	Intermediate
	Abnormal	No	71	
				
V0	Normal	_	43	Normal
				
17.1	Normal	_	53	Normal
				
13.4	Normal	_	52	Normal
				
17.2	Normal	_	N/S^a^	Normal

^a ^No pollen viability was studied in this plant.

**Figure 3:**
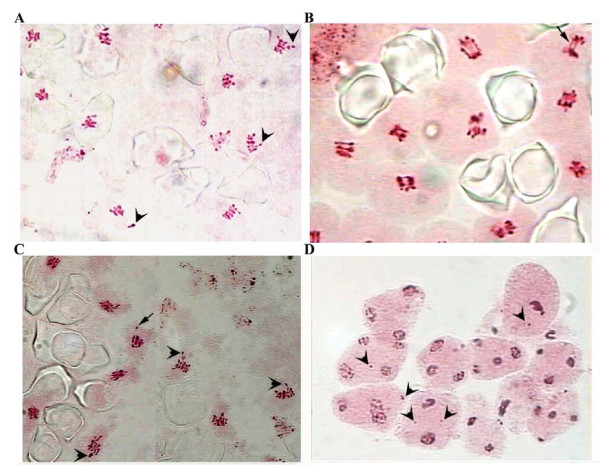
**Meiosis in *S. ruiz-lealii***. A, metaphase I showing heteromorphic bivalents (arrowheads). B, chromosomal bridges in metaphase-anaphase I (arrow). C, metaphase I with heteromorphic bivalents outside the equatorial plate (arrowheads) and early separation of univalent (arrow). D, meiocytes at telophase I and telophase II with one or two lagging chromosomes (arrowheads).

Meiotic abnormalities were observed in the two plants, with abnormal flower phenotype, that were cytologically analyzed, such as heteromorphic bivalents (Figure 3A), bridges and univalents (1 to 3 per cell) scattered outside the equatorial plate at metaphase I (Figure 3B and 3C). Many of the univalents observed at metaphase I remained as lagging chromosomes at anaphase I. At anaphase-telophase II, 16% of the meiocytes observed had between 1 and 2 laggard chromosomes per cell (Figure 3D).

### Mitochondrial genome analysis

**Figure 4:**
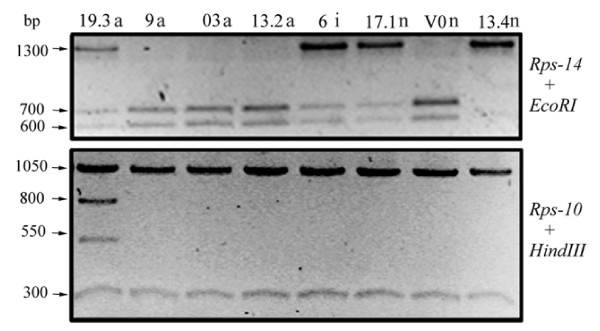
**RFLP analysis of mtDNA of *S. ruiz-lealii *plants with normal and abnormal phenotypes**. Polymorphic mitochondrial sequences. The molecular weights of bands are indicated. a, i and n, abnormal, intermediate and normal flower phenotypes, respectively, of the analyzed plants.

Mitochondrial DNA of normal and abnormal plants was analyzed to assess whether the latter shared the same mitochondrial genotype. By examining five mitochondrial sequences through PCR amplification and digestion with restriction enzymes of amplified products, it was found that only genes *rps14 *(digested with *EcoR*I) and *rps10 *(digested with *Hind*III) were informative (Figure 4); the other sequences were not polymorphic among plants. Based on the mitochondrial RFLP patterns of *rps14*/*EcoR*I and *rps10*/*Hind*III, plants were grouped according to four different mitochondrial genotypes (Table 2). Based on this analysis, abnormal plant 19.3 and normal plant 13.4 had the mitochondrial A and D genotypes, respectively. The other plants shared the B or C genotypes.

**Table 2:** Amplifications and RFLP patterns of fragments amplified from *S. ruiz-lealii *plants with five primer pairs for mtDNA.

		Plants of *S. ruiz lealii*^a^							
		
Sequence	Enzyme	19.3-a	9-a	03-a	13.2-a	6-i	17.1-n	V0-n	13.4-n
Cob		1^b^	1	1	1	1	1	1	1
	*BamH*I	2	2	2	2	2	2	2	2
	*EcoR*I	3	3	3	3	3	3	3	3
	*Hind*III	4	4	4	4	4	4	4	4
									
Rps-14		1	1	1	1	1	1	1	1
	*BamH*I	5	5	5	5	5	5	5	5
	*EcoR*I	2	3	3	3	2	2	3	4
	*Hind*III	6	6	6	6	6	6	6	6
									
Rps-10		1	1	1	1	1	1	1	1
	*Hind*III	2	3	3	3	3	3	3	3
	*Sal*I	4	4	4	4	4	4	4	4
									
Mat-r		1	1	1	1	1	1	1	1
	*EcoR*I	2	2	2	2	2	2	2	2
	*Pst*I	3	3	3	3	3	3	3	3
									
ATP 9		1	1	1	1	1	1	1	1
	*Xho*I	1	1	1	1	1	1	1	1
									
Mitochondrion type		A	B	B	B	C	C	B	D

^a ^a, i, n, abnormal, intermediate and normal flower phenotype, respectively.

^b ^1 to 6 are codes for different band patterns.

### Inheritance of abnormal flower phenotype

**Figure 5:**
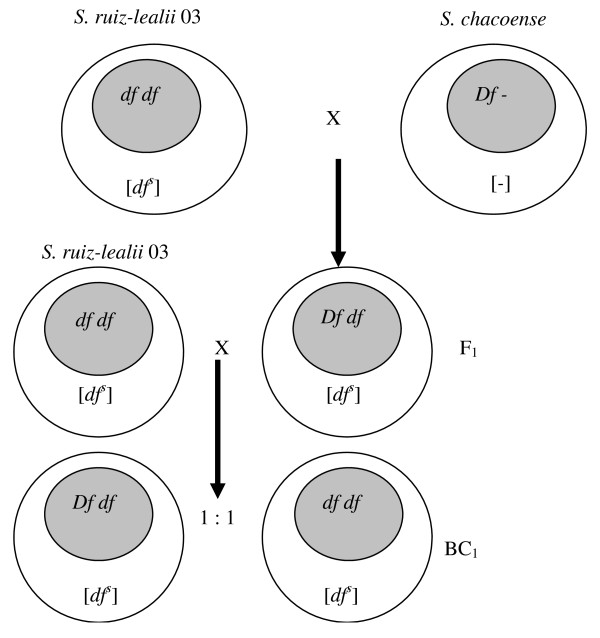
**Model of gene-cytoplasmic interaction in *S. ruiz-lealii***. Nuclear restorer genes (*Df*) in *Solanum chacoense*. The nuclear recessive (*df*) gene conditions malformed flowers in interaction with the sensible [*df*^*s*^] cytoplasm.

Only normal flowers were observed in five F_1 _interspecific hybrids obtained by crossing genotype 03 of *S. ruiz-lealii *with abnormal flower phenotype with a *S. chacoense *genotype of accession ClAlo 943 with normal flower phenotype. This could be explained by assuming that (a) the abnormal flower phenotype was the result of interactions between a recessive nuclear gene that, as an example and following Grun's terminology [4], could be designated as *df *(for *deformed flowers*), and a sensitive male sterile cytoplasmic factor, [*df*^*s*^], and (b) the *S. chacoense *genotype carried a nuclear restorer gene, *Df*.; therefore, the genotypic constitution of the F_1 _would be [*df*^*s*^] *Df df*. To confirm the presence of a sensitive [*df*^*s*^] plasmon in *S. ruiz-lealii *and the restorer *Df *gene in the genome of *S. chacoense*, a cross was performed between *S. ruiz-lealii *plant 03 and the F_1_. Under the previous hypothesis, the backcross progeny was expected to segregate 1 [*df*^*s*^] *Df df *(normal flower phenotype): 1 [*df*^*s*^] *df df *(abnormal flower phenotype) (see Figure 5). The backcross progeny did not fit the expected ratio; the segregation was distorted to the normal phenotype since 18 of the 26 evaluated plants exhibited the normal flower phenotype. Of the eight remaining plants, five were classified as having abnormal and three as having intermediate flower phenotypes. Because plants with intermediate phenotype presented normal anthers, they were grouped with the normal phenotypes for the *X*^2 ^test. In this analysis, the probability of the null hypothesis for 1:1 ratio was P = 0.001. The data presented here indicate that, with the number of plants analyzed, the segregation of the character abnormal flower in *S. ruiz-lealii *does not fit to a single Mendelian gene inheritance. A larger number of progeny would be required to conclude in this respect. However, the difficulties experienced in the crossing work due to the presence of pre-zygotic barriers prevented us for increasing the number of examined plants.

### AFLP analysis

**Figure 6:**
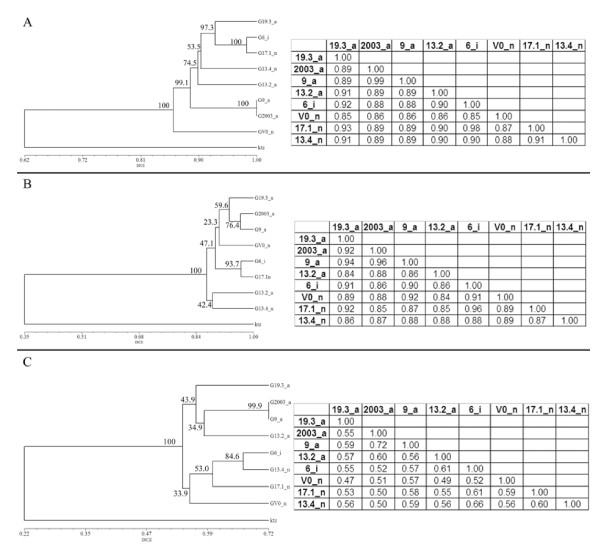
**Cluster analysis based on molecular markers of eight *S. ruiz-lealii *plants**. Dice similarity matrices and dendrograms obtained by cluster analysis based on presence/absence of AFLP (A), MSAP – methylation insensitive polymorphism (B) and MSAP – methylation sensitive polymorphism (C). a, i and n, abnormal, intermediate and normal flower phenotypes, respectively, of the analyzed plants.

The AFLP analysis was used to explore the genetic diversity in eight plants and to examine if there was correlation between flower phenotypes and the genetic variability that could be eventually detected. The analysis with eight pairs of primers produced 609 amplified fragments of which 374 (61%) were monomorphic. The percentage of genetic variability among these plants varied between 1% and 15% (Figure 6A). For example, two plants, 9 and 03, shared 607 out of 609 fragments analyzed. In the cluster analysis, plant V0 separated from the other seven, which constituted a group with a genetic similarity of above 88% (Figure 6A). In the phenetic analysis, in which plants with normal and abnormal flower phenotypes grouped together with moderate to high bootstrap support, no correlation between nDNA variability and flower phenotype was detected.

### Methylation analysis

**Figure 7:**
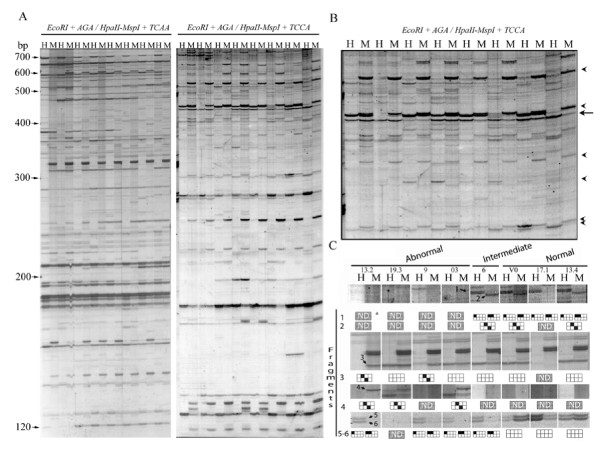
**MSAP analysis of eight *S. ruiz-lealii *plants**. A, representative MSAP profiles of two *EcoR*I/*Hpa*II (H) and *EcoR*I/*Msp*I (M) digest of DNA extracted from eight *S. ruiz-lealii *plants. The primer combinations used were E-AGA/HM-TCAA (left panel) and E-AGA/HM-TCCA (right panel). The arrows indicate positions of size markers. B, detail of the primer combination E-AGA/HM-TCCA. Arrows heads, fragments analyzed as "methylation sensitive polymorphism". Arrow, fragment analyzed as "methylation insensitive polymorphism". C, graphical interpretation of methylation sensitive fragments. The boxes represent the double-stranded recognition site (CCGG) of the *Hpa*II-*Msp*I isoschizomer. Black boxes indicate methylated cytosine. Fragments 1 and 2 epialleles present in plants with normal and intermediate flower phenotype. Fragments 3 and 4, specific epialleles of plants with abnormal flower phenotype. Fragments 5 and 6, methylated epialleles present in three plants with abnormal flower phenotype and in plant 6, with intermediate flower phenotype; and demethylated epialleles specific of plants with normal flower phenotype. ^a^Methylation patterns not determined, because the absence of a MSAP fragment can result from either a full methylation of cytosines on both strands or the absence of the restriction sites.

To test if the methylation patterns were correlated with flower abnormalities, the global methylation status of the same eight genotypes was analyzed. For MSAP analysis, six pairs of primers were used and a total of 338 fragments were analyzed (Figure 7). The MSAP bands were separated as methylation-sensitive and methylation-insensitive, to ensure that the scored epigenetic polymorphism was due to alterations in methylation and not to genetic changes at the CCGG sites.

One-hundred and seven fragments (31%) did not show differences in digestibility in *Hpa*II and *Msp*I. The presence of fragments is an indication of non-methylated CCGG restriction sites whereas their absence could be due to either variations in the CCGG nucleotide sequences or their full methylation. Anyway, these were considered as "methylation-insensitive polymorphisms" and were used, as the AFLP analysis, to survey the genetic diversity among the studied plants. Of the 107 fragments analyzed, 68 (63%) were monomorphic. The genetic variability among the studied plants varied between 4% and 16% (Figure 6B).

On the other hand, 231 (69%) of the 338 MSAP fragments, which differed in presence/absence of *EcoR*I/*Hpa*II and *EcoR*I/*Msp*I patterns in at least one genotype, were considered as "methylation-sensitive polymorphisms" and were used to estimate the epigenetic variability.

Thirty-three fragments (14%) were monomorphic in the eight plants analyzed. Twenty-five of them were present in the amplification from the *Msp*I digest, but absent from the *Hpa*II digest. The remaining fragments were present in the *Hpa*II digest, but absent in the *Msp*I digest.

The epigenetic variability detected by the "methylation-sensitive polymorphism" analysis was higher that the genetic variability detected by the AFLP analysis and also by the "methylation insensitive polymorphism" analysis. The variability in the methylation of the CCGG sequences among the plants studied varied between 28% and 53% (Figure 6B). In fact, genotype pairs such as 9-03 and 6–17.1, with 96–99% and 96–98% of genetic similarity (GS), had 72% and 61% of epigenetic similarity (ES), respectively.

In the cluster analysis, based on the presence/absence of *EcoR*I/*Hpa*II and *EcoR*I/*Msp*I fragments, plants were arranged into two groups. One group contained plants with abnormal flower phenotypes with 43.9 bootstrap value, whilst the second included plants with normal flower phenotype plus plant 6, with intermediate flower phenotype, with 33.9 bootstrap value (Figure 6C).

**Figure 8:**
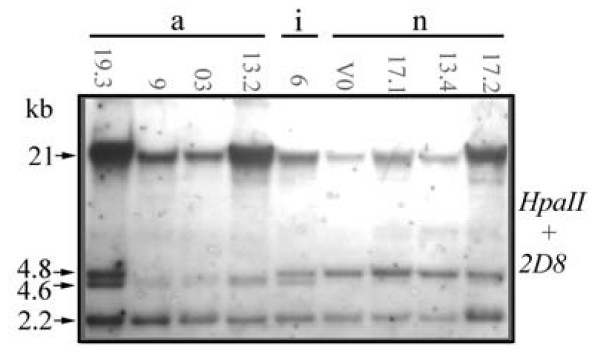
**Southern analysis of nine *S. ruiz-lealii *plants**. DNA was digested with *Hpa*II and probed with the 0.6 kb PCR product of the repetitive sequence 2D8. The molecular weights of fragments are indicated. a, i and n, abnormal, intermediate and normal flower phenotypes, respectively, of the analyzed plants.

In the analysis of the repetitive sequence 2D8, plant 17.2, which develops normal flowers as previously described, was included. Using the enzyme *Hpa*II, polymorphisms were observed for three fragments of 4.8, 4.6 and 2.2 kb (Figure 8). The smallest fragment was shared by all plants. The 4.8 kb fragment was present in abnormal plant 19.3, in the intermediate plant 6 and in all normal plants. However, the 4.6 kb fragment was found exclusively in plants with some type of flower abnormality, since it was only seen in abnormal plants 19.3, 9, 03, 13.2 and in the intermediate plant 6. Digesting the samples 19.3, 9, 03, 13.2, V0, 17.1 and 13.4 with the *Msp*I enzyme (see Materials and Methods), only a monomorphic fragment of 4.6 kb was observed (data not shown), showing that this fragment is originated from the digestion of a CCGG site present in the fragment of 4.8 kb. In addition, the presence of the 4.6 kb fragment in all the samples digested with *Msp*I, confirms that the polymorphism observed with *Hpa*II is epigenetic.

### Demethylation of a plant with normal flower phenotype

In the first season, three tubers of the normal plant 13.4 were treated (13.4T-1, -2 and -3). The three plants derived from the treated tubers grew normally but one of them did not flower. The two plants that flowered, 13.4T-1 and -2, developed seven and nine inflorescences, respectively. Six to 15 flowers per inflorescences were analyzed. Only one flower of plant 13.4T-1 was different from the flowers of the untreated control plant, 13.4. In this flower, homeotic transformations (one sepal transformed into a petal) and one leaf joined to the receptacle were observed. Petals, stamens and the gyneceum were normal. Plant 13.4T-2 produced only normal flowers. The treated plants exhibited no other morphological differences respect to the untreated control and tuberized normally.

**Figure 9:**
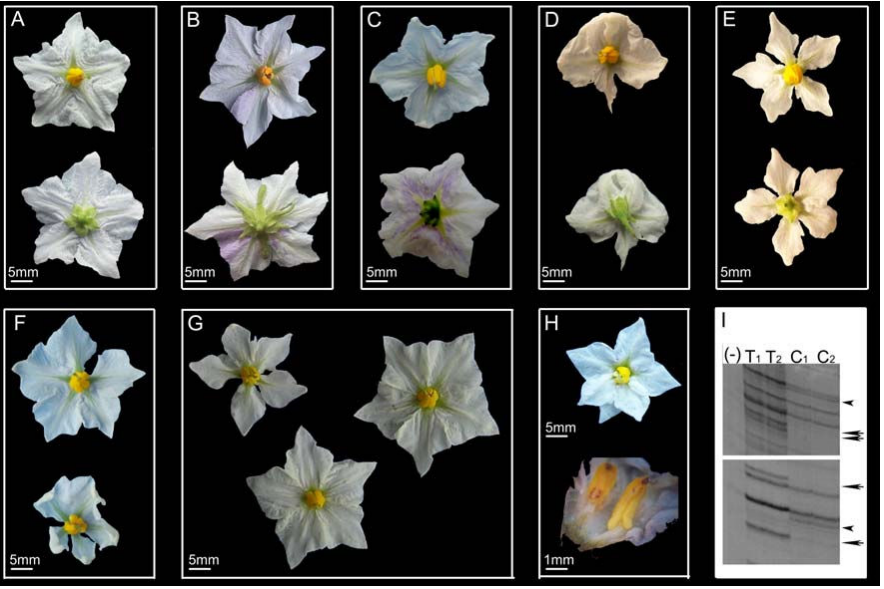
**Novel flower phenotypes observed in a *S. ruiz-lealii *plant treated with the demethylating agent AzaC**. A, flower from an untreated control plant. B, flower with overdeveloped sepals and light purple petals. C, flower with fused sepals and purple petals. D, flower with fused sepals and fused and dissected petals. E, flower with dissected petals and twisted anthers. F, two flowers from a single inflorescence, both with dissected petals. G, three flowers from a single inflorescence, two of which were normal and the remaining one presented dissected petals. H, two flowers from a single inflorescence. Upper, flower with dissected and extra number petals and twisted anthers. Lower, detail of a flower with fused stamens and petals. I, MSAP patterns observed in two independent experiments of the treated plant (T1 and T2) respect to the control untreated plant (C1 and C2). Arrowheads, hypermethylation of the treated plant respect to the control plant. Arrows, demethylation of the treated plant respect to the control plant. (-), control of the PCR experiment.

In the second season, the experiment was repeated using both, tubers of the treated 13.4T-1 plant and tubers of the control plant harvested in the previous season. One treated plant had flower phenotypes not observed in the control plants. Abnormal and normal areas for flower phenotype appeared on the same plant and only six out of 16 inflorescences presented only normal flowers. In the ten remaining inflorescences, new flower phenotypes were observed respect to the control plants. The phenotypic novelties observed were: a) change in corolla color, with light purple in the abaxial side of the petals of some flowers (Figure 9B–C); b) flowers with dissected petals that resembled plants with abnormal flower phenotype (Figure 9E–G); c) flowers with twisted anthers (Figure 9H); d) organ fusion: fused sepals, fused petals, anther-style fusion, and petal-anther fusion (Figure 9D and 9H); e) flowers with twisted and bifurcated style (not shown); f) flowers with longer sepals than the control (Figure 9B). In the control plants, a total of 58 inflorescences were evaluated, which developed only normal flowers. In addition, the 13.4 plant is maintained by vegetative reproduction and only normal flowers have been observed since 2002 (when it was obtained) to date.

To verify that the azaC affected genome methylation in treated genotypes, the MSAP patterns were compared in treated and control plants. Differences in banding patterns were observed (Figure 9I): i) fragments present in the treated plant but absent in the control plant and ii) fragments present in the control plant but absent in the treated. These results indicate that the treated plant had altered methylation patterns respect to the control plant. The hypermethylations observed could be explained by the fact that the genome of *Solanum *is able to respond to chemical treatment with azaC.

## Discussion

In a previous work, genetic and epigenetic changes were reported in synthetic diploid hybrids obtained by artificial crosses between a haploid of *S. tuberosum *and the wild potato species *S. kurtzianum *[27]. Those synthetic hybrids presented flower abnormalities such as dissected petals, atrophied anthers and homeotic transformations. In this study, we report similar flower abnormalities associated with epigenetic polymorphism in plants of *S. ruiz-lealii*. This is another wild diploid potato species of hybrid origin, as supported by the results of Raimondi *et al. *[3] and the low pollen fertility and meiotic abnormalities reported herein. These meiotic abnormalities are not related to the reported flower abnormalities because they were observed in both plants with normal and with abnormal flower phenotypes [3].

The phenotypic flower alterations observed could have their origin in genomic instabilities, as a result of putative incompatibilities between nuclear genes, or between nuclear and cytoplasmic (mitochondrial) genes. Among the mechanisms that could account for the phenotypic defects in plants of this population, point mutations in specific genes can be ruled out, because normal and abnormal flowers were observed in the same plants at the same developmental stage. No correlation between flower phenotype and nDNA variability was observed, as revealed by the AFLP analysis, in which plants with normal and abnormal flower phenotypes were grouped together with high bootstrap values (see plants 19.3, 6 and 17.1 in Figure 6A). Similar results were obtained by analyzing the methylation insensitive polymorphism. However, it is still possible that these analyses did not detect nuclear mutation responsible for the flower abnormalities.

The variability in flower phenotypes could also be attributed to changes in gene expression. Differences in gene expression can be established both, between individuals of the same progeny or population, and/or between different cells of the same tissue or organ. Thus, the presence of normal and abnormal flowers in the same plant could be interpreted as quantitative variations in a character, which show incomplete penetrance and/or variable expressivity. Reduced penetrance and variable expressivity are defined as a combination of genetic and environmental factors that influence the effects of particular genetic changes. Most of these factors have not been identified, but it is possible that differences in penetrance and expressivity would turn out to be the result of epigenetic variability.

Flower abnormalities could originate by epigenetic changes. *Arabidopsis *mutants at the DDM1 and DDM2 loci have a reduced overall level of cytosine methylation and display a number of developmental defects [29]. Transgenic *Arabidopsis *plants expressing an antisense cytosine methyltransferase RNA also exhibit abnormalities including a number of flower defects resembling the phenotypes of known flower homeotic mutants [19,20]. Two different flower regulatory genes, *SUPERMAN *and *AGAMOUS *have been found to be hypermethylated in the antisense-MET1 *Arabidopsis *plants [16]. These experiments suggest a direct cause and effect relation between DNA methylation and proper regulation of developmentally important genes. Our results of the MSAP analysis indicate that plants with abnormal flower phenotype had a similar global status of cytosine methylation, which was different from the status of the plants with normal flower phenotype. This differential methylation status between normal and abnormal phenotypes was confirmed by studying the methylation of a pericentromeric repetitive sequence, where we found that plants with (at least some) abnormal flowers shared one epiallele absent in plants with normal flowers. In order to obtain more data about the participation of DNA methylation in the origin of flower abnormalities, we reproduced the flower abnormalities observed in nature by altering chemically the methylation patterns of a plant with normal flower phenotype. In this way, a connection between DNA methylation and flower phenotype is strongly suggested by the data. Altogether, these results are consistent with the epigenetic basis of flower abnormalities in the hybrid *S. ruiz-lealii*.

Differences in methylation levels can lead to differences in gene expression, and can include variation in transcriptional levels that confer phenotypic effects [20,30]. Remodeling of DNA methylation and phenotypic and transcriptional changes have been reported in unstable genomes, as in synthetic *Arabidopsis *allotetraploids [23]. In a recent study, we showed that interspecific *Solanum *hybrids and their BC_1_progenies presented changes in methylation patterns [27]. In addition we observed flower abnormalities in those hybrids similar to the ones in *S. ruiz-lealii*. Also, in another natural diploid hybrid, *S. *× *rechei*, we observed the same type of abnormalities (unpublished results). Changes in DNA methylation induced by hybridization and/or polyploidization were reported in *Brassica*, *Triticum*, *Oryza *and *Spartina *[13,31]. Our results indicate that these observations could be extended to the tuber-bearing *Solanum*, in which case, interspecific hybridization among potato species triggers genetic and epigenetic changes that induce phenotypic alterations, affecting principally the regulation of flower development. This raises the possibility that DNA methylation may normally play a role in the regulation of flower development genes in interspecific hybrids, and that the differences in methylation can cause misregulation of this system.

However, we cannot rule out the possibility of cytoplasmic-nuclear interactions. Homeotic-like flower morphologies in plants with cytoplasmic male sterility (CMS) are maternally inherited and associated with rearrangements in the mitochondrial DNA [8]. Based on our analysis of the mitochondrial sequences, we could not detect any pattern shared exclusively by plants with abnormal flower phenotypes, which could have been linked to the presence of a mutation in the mtDNA responsible for CMS. It is also possible that normal and abnormal plants sharing the same mitochondrion could have different nuclear complements. Cytoplasmic homeosis affects flower morphology via altering the expression of homeotic genes [for review see [7]]. However, detailed mechanisms of cytoplasmic homeosis are still unclear. Our hypothesis to integrate flower development with a particular mtDNA composition is that mitochondrial dysfunction could induce epigenetic changes affecting the transcriptional activity of homeotic genes. Bereterbide *et al. *[32] studied the fusion of the stamens with the pistil in *Nicotiana tabacum *with the cytoplasm of *N. repanda*. They showed that the phenotype was partially restored by ectopic expression of the *Arabidopsis SUPERMAN *gene and that the expression of the putative tobacco *SUPERMAN *gene was significantly lower in an alloplasmic male sterile line compared with the male fertile tobacco line. These authors discussed that the impairment in gene expression might result from an epigenetic modification of Nt*SUP *sequence.

The plants used in our study are genetically closed as revealed by the AFLP analysis and confirmed by the methylation insensitive polymorphism analysis. On the other hand, we report an important level of natural variation in methylation profiles of anonymous CCGG restriction sites. Similarly, Keyte *et al. *[33] exploring the methylation polymorphism at CCGG sites in 20 accessions of cotton found a high level of methylation polymorphism that exceeded the polymorphism obtained with RFLP markers. To the best of our knowledge, this is the first work where the global methylation variability is evaluated in a tuber-bearing species of the genus *Solanum*. Interestingly, the epigenetic variability found in the natural plant population used in this study would be associated with a particular phenotype with evolutionary significance. There is an important reproductive aspect to be considered that is strongly related to flower abnormalities and the role in sexual isolation. Bumblebees, the only insects that pollinate tuber-bearing *Solanum *species, do no visit plants with aberrant flowers [34] that are, consequently, isolated from the breeding point of view. Comparing genetic and epigenetic (methylation) variability, we found that plants that presented less than 4% of genetic variability (plants 9 and 03) had a divergence in the methylation patterns of about 28%. This suggests that related plants may begin to differentiate first in their methylation patterns. Also, we observed a wide difference between genetic and epigenetic variability in plants 17.1 and 6. The flower morphology of these plants was also different. Plant 6 had rotate normal flowers and some flowers with the abnormalities described. On the other hand, plant 17.1 presented only normal stellate flowers, instead of rotate. The methylation variability among these genetically related genotypes could explain the phenotype differences observed.

In a similar study, Cervera *et al. *[35] found 24–34% of differences in the methylation of the CCGG sites among different ecotypes of *Arabidopsis thaliana*. However they found a minimal variation (less than 1%) when comparing the methylation patterns among plants of the same ecotype. In our study, we can consider that the plants of *S. ruiz-lealii *examined belong to the same ecotype, since they were collected in the same area and the morphology of the plants was similar except for the flower abnormalities described [3]. In contrast to the results with *Arabidopsis*, we found 53% of intra-ecotype methylation changes. This source of variability, unexplored in the genus *Solanum*, could indicate higher plasticity in the *Solanum *versus the *Arabidopsis *genome. In this sense, Salmon *et al. *[36] to explain the phenotypic variability reported in *Brassica *species proposed that the high methylation level and polymorphism founded in this species could be related with the high structural genome plasticity.

## Conclusion

This work contributes to extend previous observations of DNA methylation changes [13,31] induced by hybridization and/or polyploidization to species of tuber-bearing *Solanum*. Analyzing the methylation status of the natural homoploid hybrid *Solanum ruiz-lealii *by MSAP technique and Southern blot, we found association between methylation patterns and abnormal flower phenotypes. Chemical demethylation of a normal plant reproduced the abnormal phenotypes observed in hybrid plants with similar methylation patterns. Furthermore, this analysis showed that the epigenetic variability was higher than the genetic variability measured by AFLP analysis. To assess the importance of the epigenetic variation in the microevolutionary process of *S. ruiz-lealii*, the inheritance and stability of the epialleles should be established. The association between flower abnormalities and epigenetic variation found in the natural population of *S. ruiz-lealii *studied is important for potato taxonomists for two reasons. The first is the origin of new morphological types better explained by epigenetic variability (i.e. plant 17.1, with stellate flowers, and plant 6, with rotate flowers, shared 96% and 61% of the AFLP and MSAP markers, respectively). The second reason relates to the origin of new species by interspecific hybridization. Flower abnormalities can act as an isolating mechanism influencing the mating among *Solanum *species and their hybrids. Because methylation changes are potentially reversible and can regulate the degree of gene expression, after the hybrids are stabilized through several generations of clonal propagation, the fertility of the incipient species could be restored.

## Methods

### Plant material

Nine plants of a wild population of *S. ruiz-lealii *were grown from tubers in a greenhouse. All of them, except plant 03, had been previously described by Raimondi *et al. *[3]. Plant 03 was collected by the authors in the year 2002. Their flower phenotypes were: normal (plants 13.4, 17.1, V0 and 17.2); intermediate (plant 6); and abnormal (plants 19.3, 9, 03 and 13.2). The main criterion to classify plants as having abnormal flower phenotype was the presence of homeotic transformations affecting anther development or the presence of rudimentary or twisted anthers. In addition, others malformations like organ fusion and dissected petals were found in plants with this phenotype. Plants classified as having intermediate phenotype presented normal anthers, but exhibited some of the following malformations: organ fusion and/or dissected and overlapping petals. During the growing season of 2002, 2003 and 2004, flowers of each plant were morphologically characterized and pollen stainability was determined in samples of five flowers, by using 1% w/v acetocarmine and counting at least 200 pollen grains in random microscopic fields.

Five interspecific hybrids were obtained by crossing plant 03 as female with one plant of accession ClAlo 943 of *S. chacoense *as male; the latter was provided by the Potato and Forages Germplasm Bank, EEA Balcarce, INTA, Argentina. Twenty-six plants were obtained by backcrossing one F_1 _interspecific hybrid as male with plant 03. Pollinations were carried out after flower emasculation.

One plant of *S. kurtzianum*, of accession SCL 4550 (2n = 2x = 24), provided by the Potato Germplasm Bank, EEA Balcarce, INTA, Argentina, was used as outgroup in the AFLP and MSAP analyses.

### Cytological analysis

Meiotic studies were performed on over 250 meiocytes of plants 13.2 and 03 to detect abnormalities that would give support to the hybrid origin of *S. ruiz-lealii*. Flower buds were fixed in a solution of ethanol-acetic acid (3:1 v/v) for 48 h at room temperature, and stained with alcohol-hydrochloric acid carmine for one week [37]. Anthers were squashed on a drop of 45% acetic acid solution in a slide and covered with a cover slip; meiocytes were observed under a light microscope.

### PCR and restriction analyses of mitochondrial sequences

DNA was extracted from leaves according to Dellaporta *et al. *[38]. After spectrophotometric measurement of DNA concentration (GeneQuant RNA/DNA Calculator, Pharmacia Biotech), DNA was diluted in 1× TE buffer to 100 ng μl^-1 ^for use in PCR analysis. Twenty five ng of total DNA were used in PCR amplification with specific primers for mtDNA [39-43]. Amplification reactions were performed in volumes of 25 μl containing 10 mM Tris-HCl pH 8.0, 50 mM KCl, 1.5 mM MgCl2, 0.01% Tween-20, 0.01% Triton X-100, 0.4 μM of each primer, 100 μM of each dNTP and 1 unit of Taq DNA Polymerase. Amplifications were performed in a PTC-100 MJ Research (Watertown, Mass.) thermocycler, programmed for a first denaturation step of 3 min at 94°C, followed by 30 thermal cycles of 1 min at 94°C, 1 min 30 s at the annealing temperature specified in Table 3, 1 min 15 s at 72°C, and a last elongation step of 7 min at 72°C. Amplification products were analyzed by electrophoresis in 1.2% agarose gels stained with ethidium bromide, directly or after digestion with some of the following restriction enzymes: *EcoR*I, *BamH*I, *Pst*I, *Hind*III, and *Sal*I. The enzymes were chosen according to previous reports of restriction sites of analyzed sequences.

**Table 3:** Primers and mtDNA sequences analyzed

Code	Primers		Annealing temperature (°C)	Reference for origin and sequence
			
	Forward	Reverse		
Mat-r	matr 4b	matr 5	55	[39]
ATP 9	pat 9-2	pat 9-1	49.5	[40]
Rps-14	rps 14-10	rps 14-8	51	[41]
Cob	1914	1913	52	[42]
Rps-10	5' rps	rps 10-8	51	[43]

### AFLP and MSAP analysis

AFLP analysis of plants was performed as described by Vos *et al. *[44]. We used *EcoR*I and *Mse*I digested DNA to generate AFLP data. A total of eight primer combinations with different specific 3 bp overhangs were used to amplify AFLP bands. The primer combinations utilized were: E-ACG/M-CAA, E-ACG/M-CAT, E-ACA/M-CAA, E-ACA/M-CAT and E-AGC/M-CAA. The amplification products were electrophoresed on 6% polyacrylamide gels and silver stained.

The methylation pattern at the 5'-CCGG sites was analyzed using the isoschizomer methylation-sensitive enzymes *Hpa*II and *Msp*I in both random genomic DNA, using the MSAP technique, and in a repetitive genomic sequence. For MSAP analysis, the protocol developed by Reyna-López *et al. *[45] and adapted by Xiong *et al. *[46] for rice was followed. This is an adaptation of the original AFLP protocol to incorporate the use of methylation-sensitive restriction enzymes. *Hpa*II is sensitive to full methylation (both strands methylated) of either cytosine but cleaves the hemimethylated external cytosine, whereas *Msp*I is sensitive only to methylation of the external cytosine [47,48]. Fragments present in the amplification from the *Msp*I digest, but absent from the *Hpa*II digest indicate full methylation of the internal cytosine. On the other hand, fragments present from the *Hpa*II digest, but absent from the *Msp*I digest show hemimethylation of the external cytosine or hemimethylation of both cytosine. In this technique the presence of the fragments in both profiles suggests the existence of a non-methylated CCGG restriction site, while the absence of these fragments in both amplifications, from the *EcoR*I/*Hpa*II and the *EcoR*I/*Msp*I digest, could be due either to variation of the CCGG nucleotide sequence or to its full methylation. Also, the methylation sensitive enzyme *Hpa*II was used in MSAP analysis to verify if the demethylating agent 5-Azacytidine affected genome methylation in treated genotypes. If the CCGG sites change its methylation levels in treated plants, new MSAP patterns should be observed in the amplification of treated plants respect to the control.

The isoschizomers *Hpa*II and *Msp*I were used as frequent cutters and *EcoR*I was used as rare cutter. The adapters for *EcoR*I were the same as those used in the AFLP protocol. The adapters for *Hpa*II-*Msp*I digest fragments were designed according to Xiong *et al. *[46]. All primers designed for the *EcoR*I fragments had the same core and enzyme specific sequence (5'-GACTGCGTACCAATTC-3'); the following combinations of three selective nucleotides were added to the basic sequence: AGA; ACC; AAA; AAC. The *EcoR*I primers were used in combination with two *Hpa*II-*Msp*I primers that bear four selective nucleotides (in italics): 5'-CATGAGTCCTGCTCGG*TCAA*-3' and 5'-CATGAGTCCTGCTCGG*TCCA*-3'. Genomic DNA (1 μg) was digested with 20 U of *EcoR*I (New England Biolabs, Ipswich, MA) in a final volume of 40 μl of the appropriate buffer for 3 h at 37°C. For the second digestion, 20 U of *Hpa*II or *Msp*I (New England Biolabs, Ipswich, MA) were used. The digested fragments were ligated to the adapters in a buffer containing 0.5 mM of DTT, 1 mM of ATP, 2 U T4 DNA ligase (New England Biolabs), and incubated at 37°C for 2 h. The preamplification was performed by using 1 μl of the ligation products and 0.2 μM of the *EcoR*I and *Hpa*II-*Msp*I primers, without the selective nucleotides, in a final volume of 50 μl containing 1× PCR buffer, 100 μM dNTP and 1 U of Taq polymerase. The PCR experiments were performed with the following program: 30 s at 72°C, 3 min at 94°C and 30 cycles consisting of 1 min at 94°C, 1 min at 56°C, 2 min at 72°C and a final extension step of 5 min at 72°C. The preamplification products were diluted 1:10 and 1 μl was used in the selective amplification reaction with the *EcoR*I and *Hpa*II-*Msp*I primers in a final volume of 20 μl. The other components were the same as the preamplification reactions. The PCR program was the same as in the AFLP protocol [38]. The amplification products were electrophoresed on 6% polyacrylamide gels and silver stained.

### Data analysis

For both AFLP and MSAP procedures, two independent amplifications were performed for each sample. Only stable and repeatable patterns were computed for analysis. Degrees of genetic similarity were estimated in two different ways. One similarity matrix was constructed scoring AFLP fragments as present (1) or absent (0). In addition, MSAP fragments that showed only common *EcoR*I/*Hpa*II and *EcoR*I/*Msp*I patterns were scored as present (1) or absent (0) in a binary matrix of "methylation-insensitive polymorphisms" (Table 4). On the other hand, amplified fragments that differed in presence/absence *EcoR*I/*Hpa*II and *EcoR*I/*Msp*I patterns in at least one genotype were considered as "methylation-sensitive polymorphisms". For each fragment, we codified the different patterns observed from 0 to 3 (Table 4), and then this codification was converted into binary matrix for presence (1) or absence (0) of the particular patterns.

**Table 4:** Graphical representation of MSAP patterns observed and interpretation of scored MSAP bands.

	Samples								
	
	X_1_			X_2_			X_3_		
	
	*Hpa*II^a^	*Msp*I	Code^b^	*Hpa*II	*Msp*I	Code	*Hpa*II	*Msp*I	Code
**Methylation insensitive polymorphism**									
Monomorphic fragments	+	+	1	+	+	1	+	+	1
									
Polymorphic fragments	-	-	0	+	+	1	-	-	0

**Methylation sensitive polymorphism**									
Monomorphic fragments	-	+	3	-	+	3	-	+	3
	+	-	2	+	-	2	+	-	2
									
Polymorphic fragments	-	+	3	-	-	0	+	+	1
	-	+	3	-	+	3	-	-	0
	+	-	2	+	+	1	-	+	3

^a ^MSAP digestion. + presence, - absence of amplified fragments.

^b ^Number used in the data analysis to identify the different patterns observed.

Pair wise comparisons were used to generate a similarity matrix based on Dice coefficient [49]: *GS *(*ij*) = 2*a*/(2*a*+*b*+*c*), where *GS *(*ij*) is the measure of genetic (or epigenetic) similarity between individuals *i *and *j*, *a *is the number of polymorphic fragments that are shared by *i *and *j*, *b *is the number of fragments present in *i *and absent in *j*, and *c *is the number of fragments present in *j *and absent in *i*. This distance, which does not treat shared band absence as identical, was chosen because absence of a MSAP fragment can result from either a full methylation of cytosines on both strands or the absence of the restriction sites. Relationships among plants based on genetic polymorphism and methylation-sensitive polymorphism similarity matrices were established based on UPGMA (Unweighted Pair-Group Method with Arithmetic averaging). Analyses were performed with the NTSYS program [50]. For bootstrapping analysis, the WinBoot program was used [51] (1000 bootstraps involving random fragment sampling with replacement).

### DNA gel blot analysis

Repetitive sequences in plant genomes are target to cytosine methylation [52]. Thus, the differential level of cytosine methylation between plants with normal and abnormal flower phenotypes can be confirmed through the analysis of methylation in this type of sequences. We studied the methylation level of a repetitive sequence 2D8 by southern blots. The clone 2D8, a 5.9 kb tandem repeat isolated from the diploid potato species *S. bulbocastanum *[53], was kindly provided by Jiming Jiang (Department of Horticulture, University of Wisconsin). Two independent digestions were performed with *Hpa*II and *Msp*I (New England Biolabs, Ipswich, MA). Fifteen μg of the digested genomic DNA was separated by agarose electrophoresis in a 0.8% gel and transferred onto Hybond N+ membranes by the alkaline method specified by the supplier (Amersham Pharmacia). The probe used was a PCR product corresponding to the subcloned fragment of clone 2D8. The 2D8 clone was digested with *Sal*I and the DNA fragments were separated by agarose gel electrophoresis. One resulting fragment, 250 bp, was subcloned into plasmid pUC19. The biotin-labelled probe was synthesized by PCR from the subcloned fragment using a 5'-end biotinylated pUC/M13 forward primer and a pUC/M13 reverse primer. The membranes were hybridized overnight at 52°C in SDS 7%, 0.5 M Na2 HPO4 pH 7.2 and 1 mM EDTA pH 8.0. Washes were done as follows: twice with 2× SSC, 0.1% SDS for 10 min at room temperature, one wash for 30 min with 1× SSC, 0.1% SDS at 50°C followed by a 30 min wash with 0.2× SSC, 0.1% SDS at 50°C. Signals were detected using the BrightStarTM BioDetectTM kit for non-isotopic detection of biotinylated DNA probes (Ambion Inc., Austin, TX). Blots were placed in a protective plastic sheet and exposed to X-ray film for 24 h.

### 5-Azacytidine treatment

If the cytosine methylation were associated with flower abnormalities, it should be possible to induce flower abnormalities by modifying the methylation pattern of a plant with otherwise normal flower phenotype. We treated normal flower phenotype plant 13.4 with the demethylating agent 5-Azacytidine (azaC) during two seasons (2006 and 2007). Treated and untreated control plants were grown to flowering and compared phenotypically. Sprouting tubers of the plant 13.4 were placed in plastic trays with sterile substrate in a chamber with a 16 h/8 h L:D photoperiod. Drops of azaC 40 μM solution (Sigma-Aldrich) were applied to the leaves of the shoot meristems during the dark period. This process was repeated during 15 days. After that time, plants were transplanted into pots and grown in a greenhouse. In the second year, the azaC treatment was repeated on tubers of the same genotype, obtained in the previous season. Control tubers of plant 13.4 were treated similarly, but water drops were placed instead of azaC solution. All plants were grown in the same conditions. DNA was isolated from leaves of treated and untreated control plants at the same time. The MSAP analysis was performed to confirm methylation changes in treated plants respect to the untreated control. Two independent experiments were designed and only repeatable fragments were scored.

## Authors' contributions

CFM, RWM and ELC designed the research. CFM performed the research and analyzed the data. CFM and RWM drafted the manuscript. ELC contributed to writing the manuscript. All authors contributed to manuscript revision and approved the final version.

## References

[B1] Brücher HE (1962). Nuevas especies de *Solanum *(Tuberarium) de la zona semiárida del NW Argentino. Revista de la Facultad de Ciencias Agrarias UNCuyo.

[B2] Hawkes JG, Hjerting JP (1969). The potatoes of Argentina, Brazil, Paraguay and Uruguay A biosystematic study.

[B3] Raimondi JP, Peralta IE, Masuelli RW, Feingold S, Camadro EL (2005). Examination of the hybrid origin of the wild potato *Solanum ruiz-lealii *Brücher. Plant Systematics and Evolution.

[B4] Grun P, Aubertin M, Radlow A (1962). Multiple differentiation of plasmons of diploid species of *Solanum*. Genetics.

[B5] Grun P (1979). Evolution of the cultivated potato: A cytoplasmic analysis. The Biology and Taxonomy of the Solanaceae.

[B6] Hanson MR, Conde MF (1985). Functioning and variation of cytoplasmic genomes: Lessons from cytoplasmic-nuclear interactions affecting male fertility in plants. International Review of Cytology.

[B7] Zubko MK (2004). Mitochondrial tuning fork in nuclear homeotic functions. Trends in Plant Science.

[B8] Hanson MR, Bentolila S (2004). Interactions of mitochondrial and nuclear genes that affect male gametophyte development. Plant Cell.

[B9] Buckner B, Hyde BB (1982). Characterization and comparison of chloroplast DNA in several *Solanum tuberosum *subspecies involved in cytoplasmic sterility. Journal Cell Biology.

[B10] Budar F, Touzet P, De Paepe R (2003). The nucleo-mitochondrial conflict in cytoplasmic male sterilities revisited. Genetica.

[B11] Schnable PS, Wise RP (1998). The molecular basis of cytoplasmic male sterility and fertility restoration. Trends in Plant Science.

[B12] Finnegan EJ, Peacock WJ, Dennis ES (2000). DNA methylation, a key regulator of plant development and other processes. Curr Opin Genet Dev.

[B13] Rapp AR, Wendel JF (2005). Epigenetics and plant evolution. New Phytologist.

[B14] Kass SU, Pruss D, Wolffe AP (1997). How does DNA methylation repress transcription. Trends Genet.

[B15] Zhang X, Shiu S, Cal A, Borevitz JO (2008). Global analysis of genetic, epigenetic and transcriptional polymorphisms in *Arabidopsis thaliana *using whole genome tiling arrays. PLoS Genet.

[B16] Jacobsen SE, Sakai H, Finnegan EJ, Cao X, Meyerowitz EM (2000). Ectopic hypermethylation of flower-specific genes in *Arabidopsis*. Current Biology.

[B17] Jacobsen SE, Meyerowitz EM (1997). Hypermethylated SUPERMAN epigenetic alleles in *Arabidopsis*. Science.

[B18] Soppe WJJ, Jacobsen SE, Alonso-Blanco C, Jackson JP, Kakutani T, Koornneef M, Peeters AJ (2000). The late flowering phenotype of *fwa *mutants is caused by gain-of-function epigenetic alleles of a homeodomain gene. Molecular Cell.

[B19] Finnegan EJ, Peacock WJ, Dennis ES (1996). Reduced DNA methylation in *Arabidopsis thaliana *results in abnormal plant development. Proc Natl Acad Sci USA.

[B20] Ronemus MJ, Galbiati M, Ticknor C, Chen J, Dellaporta SL (1996). Demethylation-Induced developmental pleiotropy in *Arabidopsis*. Science.

[B21] Cubas P, Vincent C, Coen E (1999). An epigenetic mutation responsible for natural variation in floral symmetry. Nature.

[B22] Manning K, Tor M, Poole M, Hong Y, Thompson AJ, King GJ, Giovannoni JJ, Seymour GB (2006). A naturally occurring epigenetic mutation in a gene encoding an SBP-box transcription factor inhibits tomato fruit ripening. Nat Genet.

[B23] Madlung A, Masuelli RW, Watson B, Reynolds SH, Davison J, Comai L (2002). Remodeling of DNA methylation and phenotypic and transcriptional changes in synthetic *Arabidopsis *allotetraploids. Plant Physiology.

[B24] Torres R (2005). Hipometilación del ADN por tratamiento químico en tres especies tuberosas de Solanum. MSc thesis.

[B25] Ross H, Horn W, Röbbelen G (1986). Potato breeding-problems and perspectives Supplement 13 to Journal of plant breeding.

[B26] Stokes TL, Kunkel BN, Richards EJ (2002). Epigenetic variation in *Arabidopsis *disease resistance. Genes & Development.

[B27] Marfil CF, Masuelli RW, Davison J, Comai L (2006). Genomic instability in *Solanum tuberosum *× *Solanum kurtzianum *interspecific hybrids. Genome.

[B28] Martienssen RA, Colot V (2001). DNA methylation and epigenetic inheritance in plants and filamentous fungi. Science.

[B29] Vongs A, Kakutani T, Martienssen RA, Richards EJ (1993). *Arabidopsis thaliana *DNA methylation mutants. Science.

[B30] Kakutani T, Munakata K, Richards EJ, Hirochika H (1999). Meiotically and mitotically stable inheritance of DNA hypomethylation induced by *ddm1 *mutation of *Arabidopsis thaliana*. Genetics.

[B31] Salmon A, Malika L, Ainouche L, Wendel JF (2005). Genetic and epigentic consequences of recent hybridization and polyploidy in *Spartina *(*Poaceae*). Mol Ecol.

[B32] Bereterbide A, Hernould M, Farbos I, Glimelius K, Mouras A (2002). Restoration of stamen development and production of functional pollen in an alloplasmic CMS tobacco line by ectopic expression of the *Arabidopsis thaliana *SUPERMAN gene. Plant J.

[B33] Keyte AL, Percifield R, Liu B, Wendel JF (2006). Infraspecific DNA methylation polymorphism in cotton (*Gossypium hirsutum *L). Journal of Heredity.

[B34] Camadro EL, Carputo D, Peloquin SJ (2004). Substitutes for genome differentiation in tuber-bearing *Solanum*: Interspecific pollen-pistil incompatibility, nuclear-cytoplasmic male sterility, and endosperm. Theor Appl Genet.

[B35] Cervera MT, Ruiz-García L, Martínez-Zapater JM (2002). Analysis of DNA methylation in *Arabidopsis thaliana *based on methylation-sensitive AFLP markers. Mol Genet Genomics.

[B36] Salmon A, Clotault J, Jenczewski E, Chable V, Manzanares-Dauleux MJ (2008). Brassica oleracea displays a high level of DNA methylation polymorphism. Plant Science.

[B37] Snow R (1963). Alcoholic-hydrocholric acid carmine as a stain of chromosomes in squash preparation. Stain Technol.

[B38] Dellaporta SL, Wood J, Hicks JB (1983). A plant DNA minipreparation: version II. Plant Molecular Biology.

[B39] Vos P, Hogers R, Bleeker M, Reijans M, Lee T van de, Hornes M, Frijters A, Pot J, Peleman J, Kuiper M, Zabeau M (1995). AFLP: a new concept for DNA fingerprinting. Nucleic Acid Research.

[B40] Bégu D, Mercado A, Farré JC, Moenne A, Holuigue L, Araya A, Jordana X (1998). Editing status of mat-r transcripts in mitochondria from two plant species: C-to-U changes occur in putative functional RT and maturase domains. Current Genetics.

[B41] Dell'Orto P, Moenne A, Graves PV, Jordana X (1993). The potato mitochondrial ATP synthase subunit 9: Gene structure, RNA editing and partial protein sequence. Plant Science.

[B42] Quiñones V, Zanlungo S, Moenne A, Gómez I, Holuigue L, Litvak S, Jordana X (1996). The r*pl5-rps14-cob *gene arrangement in *Solanum tuberosum*:*rps14 *is a transcribed and unedited pseudogene. Plant Molecular Biology.

[B43] Zanlungo S, Bégu D, Quiñones V, Araya A, Jordana X (1993). RNA editing of apocytochrome b (*cob*) transcripts in mitochondria from two genera of plants. Current Genetics.

[B44] Zanlungo S, Quiñones V, Moenne A, Holuigue L, Jordana X (1995). Splicing and editing of *rps10 *transcripts in potato mitochondria. Current Genetics.

[B45] Reyna-López GE, Simpson J, Ruiz-Herrera J (1997). Differences in DNA methylation patterns are detectable during the dimorphic transition of fungi by amplification of restriction polymorphisms. Mol Gen Genet.

[B46] Xiong LZ, Xu CG, Saghai Maroof MA, Zhang Q (1999). Patterns of cytosine methylation in an elite rice hybrid and its parental lines, detected by a methylation-sensitive amplification polymorphism technique. Mol Gen Genet.

[B47] McClelland M, Nelson M, Raschke E (1994). Effect of site-specific modification on restriction endonucleases and DNA modification methyltransferases. Nucleic Acids Research.

[B48] Roberts RJ, Macelis D (2001). REBASE – restriction enzymes and methylases. Nucleic Acids Research.

[B49] Sneath PHA, Sokal RR (1973). The principles and practice of numerical classification.

[B50] Rohlf FJ (1992). NTSYS-pc numerical taxanomy and multivariate system.

[B51] Yap IV, Nelson RJ (1996). WinBoot: A program for performing bootstrap analysis of binary data to determine the confidence limits of UPGMA-based dendrograms.

[B52] Rabinowicz PD, Schutz K, Dedhia N, Yordan C, Parnell LD, Stein L, McCombie WR, Martienssen RA (1999). Differential methylation of genes and retrotransposons facilitates shotgun sequencing of the maize genome. Nat Genet.

[B53] Stupar RM, Song J, Tek AL, Cheng Z, Dong F, Jiang J (2002). Highly condensed potato pericentromeric heterochromatin contains rDNA-related tandem repeats. Genetics.

